# Validation of the Mental Illness: Clinicians’ Attitudes Scale: The Factor Structure and Psychometric Properties of the Brazilian Version

**DOI:** 10.3390/healthcare12222265

**Published:** 2024-11-13

**Authors:** Raquel Helena Hernandez Fernandes, Marcos Sanches, Sireesha Jennifer Bobbili, Simone de Godoy, Álvaro Francisco Lopes de Sousa, Pedro González-Ângulo, Kelly Graziani Giacchero Vedana, Carla Aparecida Arena Ventura

**Affiliations:** 1 PAHO/WHO Collaborating Centre for Nursing Research Development-Brazil, University of São Paulo at Ribeirão Preto College of Nursing, Ribeirão Preto 14040-902, São Paulo, Brazil; raquelhhfernandes@gmail.com (R.H.H.F.); sig@eerp.usp.br (S.d.G.); kellygiacchero@eerp.usp.br (K.G.G.V.); 2 Centre for Addiction & Mental Health, Toronto, ON M6J 1H4, Canada; marcos.sanches@camh.ca (M.S.); sireeshabobbili@gmail.com (S.J.B.); 3 Instituto de Ensino e Pesquisa, Hospital Sírio-Libanês, São Paulo 01308-060, São Paulo, Brazil; sousa.alvaromd@gmail.com; 4 División Académica Multidisciplinaria de Jalpa Méndez, Universidad Juárez Autónoma de Tabasco, Jalpa de Méndez 86040, Tabasco, Mexico; pedrogonzalez8203@gmail.com

**Keywords:** instrument validation, MICA-4, health professionals

## Abstract

Background/Objectives: In the literature, few instruments have been identified to measure the stigma of health professionals toward people with mental illness. In Brazil, until 2021, the literature did not indicate the validation of an instrument or the construction of an instrument for this purpose. Considering this gap, this study aimed to validate and estimate the reliability of the Mental Illness: Clinicians’ Attitudes Scale, version 4 (MICA-4) for the Brazilian context, examining the psychometric properties through the analysis of its internal consistency and factor structure. Methods: Psychometric testing was completed in a sample of health professionals from Primary HealthCare Units. Reliability analysis was conducted in SPSS v23. Cronbach’s Alpha and item total correlation were used. The dimensionality of the MICA was explored using exploratory factor analysis (EFA) in Mplus 8.2. Results: A total of 195 health professionals participated in the research. Cronbach’s Alpha was 0.68 and according to the reliability analysis, items 10 and 12 of the original version were deleted, resulting, therefore, in 14 items. In addition, we demonstrated that it is possible to have only two factors instead of five factors, which is the number of factors in the original version of the MICA-4. Conclusions: This validated instrument for the Brazilian context can serve as an important tool in understanding the phenomenon of the stigma of health professionals toward people with mental illness and, consequently, in promoting anti-stigma strategies in Brazil.

## 1. Introduction

People with mental illness are commonly considered as a burden to society, as well as weak and unworthy of sympathy or empathy [1,2,3]. These beliefs are considered to be social constructions that lead to stigma, negatively labelling individuals with mental suffering and thus causing their devaluation. This devaluation stems from the perspective of abnormality held toward mental illnesses and their consequences. Consequently, individuals with mental illnesses are negatively singled out due to being labeled as abnormal [4].

Stigma can occur on several levels and can involve a combination of stereotypes (the association of difference with negative beliefs), prejudice (negative attitudes), and discrimination (the consequences of attitudes and prejudices against the labeled individual) [5,6]. In this sense, stigma can be present in policies and laws that exclude people with mental illness, creating structural stigma. In this scenario, policies and laws tend not to protect the rights of people with mental illness, opening possibilities for rights violations. In addition, it can be present in the attitudes and behaviors that cause social distancing and the loss of status in people with mental illness, known as interpersonal stigma. In this case, friends, family, and society, in general, distance themselves from people with mental illness, causing damage to relationships with these people. Finally, it can lead to self-stigma, which is when the stigmatized person incorporates the stigma they suffer. Self-stigma can cause low self-esteem, poor adherence to treatments, and hopelessness [7].

Studies have shown that self-stigma has a direct impact on lowering self-esteem, contributing to the formation of negative self-perceptions [8]. It is important to highlight that self-stigma has a direct effect on reducing treatment adherence and, indirectly, causes negative psychological effects such as low self-esteem, a lack of self-efficacy, demoralization, hopelessness, depression, and a negative perception of the beneficial effects of treatment. These direct and indirect impacts were observed in studies that found that people who avoid being labeled, such as those with mental disorders, tend to avoid negative emotional reactions, which ultimately reduces their participation and adherence to treatments [7,8,9,10,11,12,13,14,15]. Furthermore, self-stigma leads people with mental disorders to feel irresponsible and incapable in the eyes of close friends and family [16].

The abandonment of treatment, poor adherence, or late seeking of care can lead to delays in diagnosis and, consequently, to the chronicity of mental illness. One of the effects of this is the increased treatment costs for the public health system, especially considering that the phenomena of stigma and self-stigma are obstacles to health promotion, which contributes to cost reduction in the aforementioned system [17].

In the economic sphere, people with mental illness also face difficulties in entering the job market, a common complaint according to the studies by Crepalde and colleagues [18] and Gasparini and colleagues [19]. In this context, they are often seen as incapable of holding a job.

Furthermore, the lack of job opportunities for these individuals contributes to economic losses, as fewer people are actively working for the benefit of society.

In the context of healthcare services, the research indicates that health professionals are equally susceptible to holding stigmatizing beliefs and displaying stigmatizing behaviors toward individuals with mental illness, just as with the general public [20,21,22]. In this sense, thinking about interpersonal stigma and self-stigma, the research indicates that the stigma perpetuated by health professionals is associated with reduced help-seeking behaviors and poor adherence to mental health treatments among individuals living with a mental illness [23,24,25], configuring barriers to accessing health services [26], which has a strong impact on mental health treatments and mental health recovery. In this way, there is a reinforcement of negative attitudes and behaviors toward people with mental illness [27].

In this respect, the study by Pinheiro and Spink [28] demonstrated that the stigma experienced by people with mental illness is reflected in a limited access to health services. The study revealed that health professionals working across multiple healthcare services in Brazil were unwilling to empathize with the complaints and suffering voiced by patients with mental illness. As a result, these professionals hindered the patients’ genuine access to certain healthcare services.

It is worrying to perceive the existence of stigma on the part of health professionals toward people with mental illness in the Brazilian context [29]. According to the Ministry of Health, 3% of the national population suffers from a severe or persistent mental illness and there is an estimate that the overall prevalence in Brazil is much higher when less severe mental illnesses are considered [30].

In Brazil, the population has the right to universal and public access to the Brazilian health system, named the Single Health System (SUS), according to the Brazilian Federal Constitution of 1988 [31]. However, mental health has been the subject of the Brazilian Psychiatric Reform since the 1970s, which is a movement motivated by the tragic past in relation to the care of people with mental illness and their exclusion from society through long-term psychiatric hospitalizations and inhumane treatments [32]. In this sense, in 1990, Brazil enforced the Declaration of Caracas and incorporated the idea that mental health is anchored in Primary Healthcare (PHC), which is the gateway to the SUS and is a level-preferred option that offers action for mental health, in addition to being a strategic point of the Psychosocial Care Network [33,34].

Health professionals can have their opinions widely disseminated [35], and in Brazil, they hold a position that enables them to act as powerful agents of destigmatization. They can promote humanized and welcoming treatment focused on the recovery of individuals with mental illness [36,37]. Given this, it is essential to investigate the stigma that may arise from these professionals.

By addressing this issue, human rights can be better protected, and mental health care resources can be improved [38]. One way to understand the phenomenon of stigma is by using psychometric instruments designed to measure it. However, few instruments have been adapted and validated to measure stigma among health professionals. In this sense, according to research conducted by Wei et al. [39], there are approximately fifteen instruments designed for health professionals.

Therefore, this study aims to investigate the applicability of the Mental Illness: Clinicians’ Attitudes Scale (MICA-4) among healthcare professionals [40] working in Primary Healthcare (PHC) settings in Brazil. Our objective is to validate and determine the reliability of the MICA-4 scale with a sample from Brazil, examining its psychometric properties by assessing its internal consistency and factor structure.

## 2. Materials and Methods

### 2.1. Background

This research is part of a randomized controlled trial, which includes a cross-sectional component, designed to explore the presence of stigma among healthcare professionals toward individuals with mental illness and substance use disorders in Brazilian PHC settings. The study is titled “Exploring Stigma, Discrimination, and Recovery-Based Perspectives toward Mental Illness and Substance Use Problems among Primary Healthcare Providers in Ribeirão Preto, Brazil: A Randomized Controlled Trial”. For the study to be developed in Brazil, it was necessary to validate three scales that measure the stigma of health professionals in relation to people with mental illness for the Brazilian context, which was the cross-sectional part. One of the validated scales for Brazil is the Opening Minds Scale for HealthCare Providers (OMS-HC) [41]. The project is being implemented in Family Health Units (FHUs), which are public health units intended for continued care with follow-up in basic specialties, with a multidisciplinary team capable of carrying out activities to promote, protect, and recover health, and characteristics of the level of primary care, through the Family Health Strategy (FHS). The FHS began with the Family Health Program (FHP), created by the Brazilian Ministry of Health in 1994. Since then, the FHP has been defined as a priority strategy for the organization and strengthening of PHC in the country. The FHP presented itself as a new way of understanding health, having the family as the center of care and going beyond the idea of treating only the sick individual [42]. The FHS increases accessibility and first-contact care by placing interdisciplinary health teams close to people’s homes, valuing the territory and the individuals belonging to the territory. As a result, these diverse teams of professionals provide the comprehensive and proactive care needed by the most vulnerable and marginalized communities [30,43,44,45]. An FHS team is composed of a nurse, a nursing assistant, a doctor, and four to six community health workers, all of them full-time workers [30].

### 2.2. Participants

Participants were health professionals from six FHUs in Ribeirão Preto, a municipality in the State of São Paulo, Brazil, who are participating in the aforementioned project. All health professionals, including community health workers, were invited. The city of Ribeirão Preto has 14 FHUs, and the units participating in this research were randomly selected. It is important to highlight that some Basic Health Units (BHUs) in the city of Ribeirão Preto are undergoing transformations and becoming FHUs, and this research was conducted during that process. Therefore, it will be possible to observe in this study whether there is mention of Basic Health Units as places of participation. Data collection with the participants took place from December 2019 to May 2020. The inclusion criterion chosen was the working time in the unit, being at least 1 month. The chosen exclusion criterion was the option to respond to only one of the instruments and not all the proposed instruments.

### 2.3. Aims

The MICA-4 has not yet been validated for the Brazilian context. Our aim with this analysis was to validate and estimate the reliability of the MICA-4 scale using a sample of subjects from Brazil.

### 2.4. Measures

The MICA-4 is a 16-item self-report questionnaire. This instrument originated from improvements to the MICA-2 and MICA-3 instruments, as the authors aimed to construct a tool for nursing students. Consequently, the authors administered the instrument to 191 nursing students from a British university [40].

The research method followed all stages of validation, resulting in a Cronbach’s Alpha value of 0.72 and a total item correlation ≥0.2, indicating good internal consistency. Pearson’s correlation coefficient was calculated, taking into account the Emotional Reactions to Mental Illness Scale (ERMIS) and the Reported and Intended Behavior Scale (RIBS), resulting in a *p*-value of <0.001 (0.3–0.5), indicating convergent validity. Moreover, concerning acceptability, all items of the MICA-4 met the criterion of only 5% missing data, indicating good acceptability. Thus, it was found that the MICA-4 is reliable, valid, has good internal consistency, and has acceptable measures for assessing stigma among healthcare students. It is important to note that the authors suggest that this scale can also measure stigma among healthcare professionals [40].

The minimum score that an individual can obtain in the answers is 16, meaning fewer stigmatizing attitudes, and a maximum of 96, meaning more stigmatizing attitudes. It is a Likert-type scale and the anchor points are 1–6 (1 = strongly agree, 2 = agree, 3 = partially agree, 4 = partially disagree, 5 = disagree, and 6 = strongly disagree). Items that require reverse coding are 1, 2, 4, 5, 6, 7, 8, 13, 14, and 15 for calculating the total score. The estimated time for the health professional to answer the scale in its entirety is 5.0 min, although in its construction, the response time was 3.7 min [40].

The MICA-4 measures five dimensions related to stigma: Visions of the Fields of Social Assistance, Health, and Mental Disorder, with corresponding items 2, 5, 8, 10, 12, 15, and 16; Knowledge about Mental Disorder, with corresponding items 1, 3, and 13; Disclosure, with corresponding items 4 and 7; Distinction between Physical and Mental Health, with corresponding items 11 and 14; and Care for the Patient with Mental Disorder, with corresponding items 6 and 9 [40,46].

### 2.5. Data

The research team performed cultural modifications to the MICA-4 for application in Brazil. The initial instrument was translated from English into Brazilian Portuguese and subsequently reviewed by a panel of experts proficient in both languages. Posteriorly, a back-translation process and a pre-test were conducted. The pre-test was carried out between July and August of 2018 in seven health units located in the Municipality of Ribeirão Preto, with two FHUs and five Basic Health Units. In total, 40 health professionals participated. The findings from the pre-test indicated that the Brazilian version of MICA-4 displayed sufficient adequacy and ease of comprehension. It also featured well-designed formatting, contributing to its user-friendly nature, while maintaining a reasonable level of consistency with the original version. [47].

The validation process for the culturally adapted MICA-4 scale commenced to confirm its psychometric properties [48,49]. Data collection for validation took place from December 2019 to May 2020, with a cross-sectional design and in-person administration by a research team previously trained to use the instrument.

Thus, data collection was carried out with health professionals and community health workers. The researchers approached the participants at times when they were not attending to the population and stood by the participants to clarify doubts during the application of the scale.

### 2.6. Statistical Analysis

The researchers conducted initial data cleaning and inspection, which involved reverse coding negatively worded items to enhance result interpretation. The majority of the items did not have any missing values. However, a few items had missing values from 1 to 4 subjects. These values were retained in the data and addressed using full maximum likelihood estimation or pairwise deletion, depending on the analysis.

Reliability analysis was conducted in SPSS v23. Cronbach’s Alpha and item-total correlation were used to evaluate how each item correlated with the total scale with the item removed. Items with a correlation lower than 0.2 were flagged for further investigation of item interpretation in the Brazilian context.

Face validity [50] was accessed by the evaluation of experts who participated in the stage of cultural adaptation to the Brazilian context. At that moment, experts disagreed with each other in relation to only a few items for modification, with a percentage of agreement for modification of 60% [47]. In this sense, the literature states that 90% should be considered an acceptable rate of agreement among committee members [51,52]. Therefore, there was no significant agreement between the experts to change the items. Thus, during the processes of translation, evaluation by the expert committee, and back-translation, it was found that the meanings of the words were maintained, even taking into account the Brazilian context, and the final version, validated for the Portuguese Brazilian version, remained as faithful as possible to the original version of MICA-4.

The convergent validity of the MICA-4 scale was assessed by calculating the Spearman correlation between the total scores of the MICA-4 and the OMS-HC [41]. The OMS-HC is a self-report questionnaire composed of 20 items that measure two dimensions of stigma: “Attitudes of healthcare providers toward people with mental illness” and “Attitudes of healthcare providers toward the disclosure of a mental illness” [53].

To explore the dimensional structure of the MICA-4, an exploratory factor analysis (EFA) was performed using Mplus 8.2 [54]. The analysis used a weighted least squares mean and variance-adjusted chi-square test (WLSMV) estimation method suitable for ordinal items and based on polychoric correlations [55]. Geomin oblique rotation [56] was applied to aid in interpreting the factor loadings. The number of factors was determined through parallel analysis, comparing eigenvalues from 1000 synthetic correlation matrices of random data (i.e., without a factor structure) to the actual eigenvalues from the study data [57].

## 3. Results

### 3.1. Sociodemographic Characteristics

The MICA-4 had a mean score of 36.8 with a standard deviation of 8.25, based on data from 195 participants. Of these, 107 (54.87%) were healthcare professionals and 88 (45.1%) were community health workers. The majority of the healthcare professionals were nurses, but the group also included managers, pharmacists, and dentists. On average, the participants were 45.0 years old (SD = 9.0), and 88.2% were female. In Table 1, it is possible to observe the characteristics of the sample and scale.

### 3.2. Reliability

Initially, the MICA-4 total score with 16 items showed reasonable reliability (Cronbach’s Alpha = 0.68). The item-total correlation was lower than 0.2 for items 10 and 12 (correlations = 0.16 and 0.11, respectively) (Table 2). They also had low estimated loadings in the exploratory factor analysis. Upon further investigation of the interpretation of the items in the Brazilian context, it was decided to remove them from the final scale. After the removal of these items, the reliability was still the same (Alpha = 0.68).

### 3.3. Validity

Face validity, as mentioned earlier, was carried out in a stage prior to this research, with expert involvement [47]. The MICA-4 showed a moderate to large association with the OMS-HC scale, which we took as reasonable evidence of convergent validity (Spearman correlation = 0.62, *p* < 0.001).

### 3.4. Dimensionality

As an initial investigation of the appropriateness of the data for an exploratory factor analysis, we estimated that the Kaiser–Meyer–Olkin criterion was 0.63 and the Bartlett’s test for sphericity (if the correlation matrix between the items is different from an identity matrix) resulted in a *p*-value < 0.0001.

The results of the EFA indicated two factors (refer to Table 2). This aligns with the number of factors indicated by both the parallel analysis and the interpretation. However, other criteria, such as an eigenvalue greater than one, suggested the existence of more than two factors. The MICA-4 scale was originally designed with five factors [40], but in the Brazilian context, the five-factor model appeared to be excessive. Based on the item interpretations, the first factor was labeled “Views on mental health care”, while the second factor was termed “Views on the person with mental illness”.

Overall, it is evident that some items are associated with more than one factor. For example, item 1 “*I just learn about mental health when I have to, and would not bother reading additional material on it*” loads on both factors with loadings that are not very different. The same happens with item 2. “*People with severe mental illness can never recover enough to have a good quality of life*”. This perhaps shows that the factor structure is not very strong. We also tried to study the relevance of using these two factors as subscales by using a bi-factor model [58,59], but we were not able to fit the bi-factor structure to the data due to a lack of convergence and inconsistent estimates. This also seems to indicate that it may be inadequate to use MICA-4 subscale scores, as they may be weak in defining truly different constructs when compared to the overall MICA-4 score.

In comparison with the available literature, we do not see a lot of overlap between the factors found, which are “Views of health/social care field and mental illness” (Factor 1), “Knowledge of mental illness” (Factor 2), “Disclosure” (Factor 3), “Distinguishing mental and physical health” (Factor 4), and “Patient care for people with mental illness” (Factor 5) [40], and the factors we found now. We have not found any other published material that looked at exploratory factor analysis for the MICA-4 scale, particularly accounting for the ordinal nature of the items as we did.

## 4. Discussion

The objective of this study was to validate and estimate the reliability, dimensionality, and structure of the MICA-4 scale using a sample of health professionals from FHUs in Brazil. This is the first study to translate, adapt, and validate the MICA-4 for the Brazilian context, with the involvement of primary care health professionals, including community health workers. The evaluation of the convergent validity of the MICA-4 in Brazil and in the context of Primary Care was carried out through correlation with the OMS-HC scale validated for Brazil [41]. There was a positive and moderate correlation between the MICA-4 and the OMS-HC, as expected. The scale also demonstrated the face and content validity in the evaluation of experts, both in the development phase and in the current assessment phase.

It is important to highlight the changes that the validation process provided for the MICA-4 validated for the Brazilian context. Therefore, in relation to the number of items, the original version of the MICA-4 has 16 items. In the version validated for the Brazilian context, according to reliability analysis, items 10 and 12 of the original version were deleted, leaving 14 items. The removal of these two items was also motivated by the fact that they seemed to be inconsistently interpreted by research participants.

The original version of the MICA-4, with 16 items, is composed of five factors related to stigma toward people with mental suffering [40]. As for the Brazilian version, we tried to demonstrate that it is possible to have only two factors, being named by the research team as “Views on mental health care” and “Views on the person with mental illness”, taking into account the grouping of items with exploratory factor analysis and what they seek to measure, even with the perception that some items fit both factors. Our study is one of the first to examine the MICA-4 scale using a two-factor model, enabling a more thorough investigation of the psychometric properties of the scale and its subscales in terms of dimensionality and reliability.

Comparing our study with others, it is important to mention that a study has been published by Vistorte and colleagues [60] that examined the psychometrics of the MICA-16 for the Brazilian population, specifically among primary healthcare physicians. They identified issues with the five-factor structure from the original study and proposed a new structure with only three subscales, based on expert evaluation of the items. Our study reached a somewhat different conclusion, leading to the removal of two items and the suggestion of only two subscales. This exploratory approach arose from our realization that the original structure was not functioning well in Brazil.

In this sense, our study appears to identify more issues with the use of the MICA-4 in Brazil, including lower reliability than what Vistorte and colleagues reported. Additionally, there are significant population differences; their study involved a more homogeneous population from a larger, more developed area of the country, whereas they used a sample that was more representative of Brazil as a whole. They also assumed that the factor structure would be the same in Brazil as in the other three Spanish-speaking samples. We believe both studies offer valuable insights and should be replicated: ours for being exploratory and theirs for not relying on a predefined factor structure.

The reliability in the Brazilian context did not prove to be high in our study, despite our use of a more homogeneous population compared to the study by Vistorte and colleagues [60], which included populations from Brazil, Bolivia, Chile, and Cuba. In Brazil, although there are cultural differences between regions, the Unified Health System is standardized, and primary healthcare professionals are consistent across healthcare units. In the case of Vistorte’s study, it can be deduced that cultural and healthcare system differences are quite distinct since the study was conducted across different countries. Furthermore, our study encompassed a broader range of primary healthcare professionals (physicians, nurses, nursing technicians, nursing assistants, dentists, dental aides, pharmacists, and pharmacy assistants), including community health agents, whereas Vistorte’s study only included primary care physicians.

We believe that the study of subscales is an important feature of our research that provides new insights, as it differs from the study that validated the MICA-4 [60] and from the original scale. The other study has the significant limitation of not reporting factor loadings or inter-item correlations, making it impossible to evaluate whether all items are functioning well within each subscale. We also find it surprising that they obtained higher reliability without conducting an exploratory factor analysis. For these reasons, we feel that our considerations regarding dimensionality and the less favorable validation results should be included in the literature as a replication study, adding caution to the use of the MICA-4 in Brazil.

Our findings provide reasonable evidence that the validated Brazilian version of the MICA-4 is reliable, valid, and suitable for assessing stigma among healthcare professionals toward individuals with mental illness.

One limitation of this study is that the sample was limited to healthcare professionals working in PHC settings in a single municipality of Brazil. It is important to highlight that Ribeirão Preto is located in one of Brazil’s most prosperous states, São Paulo. The city has a Human Development Index (HDI) of 0.800, while the state of São Paulo has an HDI of 0.806 [61], with both classified as high. In terms of Gross Domestic Product (GDP), Ribeirão Preto registers a value of BRL 55,484.91, while the GDP of the state of São Paulo reaches BRL 2,719,751.00 [61], figures that reflect the region’s solid quality of life. In this sense, the results of this study may not be the same for health professionals who work in services other than primary care and are located in other parts of the country due to the heterogeneity of the Brazilian population.

Additionally, a limitation to consider is the predominance of female participants, which can be explained by the greater presence of professionals of this gender in FHUs. This is largely due to the composition of the teams, which are predominantly made up of nurses, nursing technicians, and nursing assistants—professions traditionally occupied by women [62].

Our sample was also not selected randomly, which could introduce some selection biases. For example, due to voluntary participation, participants with a higher degree of stigma might be less likely to agree to participate. There could also be differential probabilities of participation related to demographics, work experience, job position, or other factors that might be important for properly representing the target population. Unfortunately, we are unable to correct for or quantify this type of bias due to a lack of information about the distribution of these variables in the target population.

## 5. Conclusions

This version of the scale can contribute to studies that aim to investigate the phenomenon of stigma in this context of health professionals in Brazil and, consequently, can contribute toward possible anti-stigma interventions, strengthening internal protocols of primary care health units for the reception of patients with mental illness and envisioning the improvement of public policies related to mental health. The use of the MICA-4, validated for the Brazilian context, can promote a cascade effect in the sense that it not only measures stigma but brings reflections with its items and factors about how stigma is perceived by health professionals, promoting a clearer picture that can be used for anti-stigma interventions. Finally, the validation of this scale will contribute to the previously mentioned randomized controlled trial, making it a powerful tool for understanding the phenomenon of stigma in the context where the study is being conducted, and thus assisting in the development of intervention proposals for PHC professionals.

## Figures and Tables

**Table 1:** Characteristics of the sample and scale.

		Min/Max	N	%
Total			195	100.0%
MICA-4	Mean (SD) = 36.8 (8.2)			
Factor 1 MICA 8 Items	16.5 (4.6)	8/31		
Factor 2 MICA 6 Items	17.9 (4.7)	6/31		
Health Service	BHU		11	5.6%
	FHU		184	94.4%
Gender	Female		172	88.2%
	Male		23	11.8%
Occupation/function in the health unit	Nurse		15	7.7%
	Nursing Assistant		39	20.0%
	Nursing Technician		11	5.6%
	Community Health Worker		88	45.1%
	Other *		42	21.5%
Specialization	Yes		51	27.7%
	No		133	72.3%
Know someone with a mental illness	Yes		177	91.2%
	No		17	8.8%
Have had personal experience with a mental illness	Yes		130	67.4%
	No		63	32.6%
Age (Mean/SD)		19.0/66.0	45.0	9.0
Questionnaire time in minutes (Mean/SD)		4.0/144.0	10	11
Time working in months (Mean/SD)		6.0/475	207	113
Time working in the health unit in months (Mean/SD)		3.0/372	83	79

* Other: it consists of the following professionals: physicians, dentists, dental aides, pharmacists, and pharmacy assistants.

**Table 2:** Standardized factor loadings for exploratory factor analysis with 2 factors estimated using the WLSMV method and GEOMIN oblique rotation.

Item Labels	Factor 1—Views on Mental HealthCare	*p*-Value	Factor 2—Views on The Person with Mental Illness	*p*-Value	Original Factors MICA-4
11. It is important that any health/social care professional supporting a person with mental illness also ensures that their physical health is assessed.	0.700 *	<0.0001	−0.028	0.6426	Factor 4 (Distinguishing mental and physical health)
3. Working in the mental health field is just as respectable as other fields of health and social care.	0.597 *	<0.0001	0.013	0.8634	Factor 2 (Knowledge of mental illness)
9. If a senior colleague instructed me to treat people with mental illness in a disrespectful manner, I would not follow their instructions.	0.536 *	<0.0001	0.06	0.4593	Factor 5 (Patient care for people with mental illness)
8. Being a health/social care professional in the area of mental health is not like being a real health/social care professional.	0.510 *	<0.0001	0.267 *	<0.0001	Factor 1 (Views of health/social care field and mental illness)
16. If a colleague told me they had a mental illness, I would still want to work with them.	0.510 *	<0.0001	0.139 *	0.0199	Factor 1 (Views of health/social care field and mental illness)
15. I would use the terms “crazy”, “nutter”, “mad”, etc., to describe to colleagues people with mental illness that I have seen in my work.	0.504 *	<0.0001	0.087	0.2232	Factor 1 (Views of health/social care field and mental illness)
13. If a person with a mental illness complained of physical symptoms (such as chest pain), I would attribute it to their mental illness.	0.416 *	<0.0001	0.059	0.3472	Factor 2 (Knowledge of mental illness)
14. General practitioners should not be expected to complete a thorough assessment of people with psychiatric symptoms because they can be referred to a psychiatrist.	0.280 *	<0.0001	0.170 *	0.0134	Factor 4 (Distinguishing mental and physical health)
1. I just learn about mental health when I have to, and would not bother reading additional material on it.	0.297 *	<0.0001	0.368 *	<0.0001	Factor 2 (Knowledge of mental illness)
2. People with severe mental illness can never recover enough to have a good quality of life.	0.250 *	<0.0001	0.291 *	<0.0001	Factor 1 (Views of health/social care field and mental illness)
7. If I had a mental illness, I would never admit this to my colleagues for fear of being treated differently.	−0.140 *	0.0009	0.834 *	<0.0001	Factor 3 (Disclosure)
5. People with mental illness are dangerous more often than not.	0.083	0.2069	0.383 *	<0.0001	Factor 1 (Views of health/social care field and mental illness)
6. Health/social care staff know more about the lives of people treated for a mental illness than do family members and friends.	0.054	0.4342	0.358 *	<0.0001	Factor 5 (Patient care for people with mental illness)
4. If I had a mental illness, I would never admit this to any of my friends because I would fear being treated differently.	−0.016	0.7558	0.705 *	<0.0001	Factor 3 (Disclosure)
10. I feel as comfortable talking to a person with mental illness as I do talking to a person with physical illness. **	-	-	-	-	Factor 1 (Views of health/social care field and mental illness)
12. The public does not need to be protected from people with mental illness. **	-	-	-	-	Factor 1 (Views of health/social care field and mental illness)

* indicates statistical significance. We focused the interpretation of the factors on the loadings that are higher than 0.3 or the loading that is the highest for the item (in bold). ** are the items that were removed for low correlation and inconsistency with the other items.

## Data Availability

The study database is on file in Google Drive and can be requested from the correct correspondent via email.
